# Co-Evolution of Somatic Variation in Primary and Metastatic Colorectal Cancer May Expand Biopsy Indications in the Molecular Era

**DOI:** 10.1371/journal.pone.0126670

**Published:** 2015-05-14

**Authors:** Richard Kim, Michael J. Schell, Jamie K. Teer, Danielle M. Greenawalt, Mingli Yang, Timothy J. Yeatman

**Affiliations:** 1 Department of Gastrointestinal Oncology, H. Lee Moffitt Cancer Center and Research Institute, Tampa, FL, United States of America; 2 Department of Biostatistics and Bioinformatics, H. Lee Moffitt Cancer Center and Research Institute, Tampa, FL, United States of America; 3 Merck Co, Inc., Boston, MA, United States of America; 4 Gibbs Cancer Center and Research Institute, Spartanburg, SC, United States of America; Sapporo Medical University, JAPAN

## Abstract

**Introduction:**

Metastasis is thought to be a clonal event whereby a single cell initiates the development of a new tumor at a distant site. However the degree to which primary and metastatic tumors differ on a molecular level remains unclear. To further evaluate these concepts, we used next generation sequencing (NGS) to assess the molecular composition of paired primary and metastatic colorectal cancer tissue specimens.

**Methods:**

468 colorectal tumor samples from a large personalized medicine initiative were assessed by targeted gene sequencing of 1,321 individual genes. Eighteen patients produced genomic profiles for 17 paired primary:metastatic (and 2 metastatic:metastatic) specimens.

**Results:**

An average of 33.3 mutations/tumor were concordant (shared) between matched samples, including common well-known genes (APC, KRAS, TP53). An average of 2.3 mutations/tumor were discordant (unshared) among paired sites. KRAS mutational status was always concordant. The overall concordance rate for mutations was 93.5%; however, nearly all (18/19 (94.7%)) paired tumors showed at least one mutational discordance. Mutations were seen in: *TTN*, the largest gene (5 discordant pairs), *ADAMTS20*, *APC*, *MACF1*, *RASA1*, *TP53*, and *WNT2* (2 discordant pairs), *SMAD2*, *SMAD3*, *SMAD4*, *FBXW7*, and 66 others (1 discordant pair).

**Conclusions:**

Whereas primary and metastatic tumors displayed little variance overall, co-evolution produced incremental mutations in both. These results suggest that while biopsy of the primary tumor alone is likely sufficient in the chemotherapy-naïve patient, additional biopsies of primary or metastatic disease may be necessary to precisely tailor therapy following chemotherapy resistance or insensitivity in order to adequately account for tumor evolution.

## Introduction

Colorectal cancer (CRC) is the third most common cancer in both men and women [1]. About one third of those patients will eventually succumb to the disease. Many patients will present with synchronous metastatic disease or eventually develop metachronous metastatic disease after the resection of the primary tumor. Recently, to better understand the disease, a genome-scale analysis was conducted in colorectal cancer by the TCGA network using tumor and normal samples [2]. In that study, as expected, *APC*, *TP53*, *KRAS*, *SMAD4*, *BRAF* and *PIK3CA* mutations were commonly found. Many additional, less frequently observed mutations were also identified. Because this study was largely limited to the analysis of primary cancers, further insight into metastatic disease was warranted.

**Fig 1:**
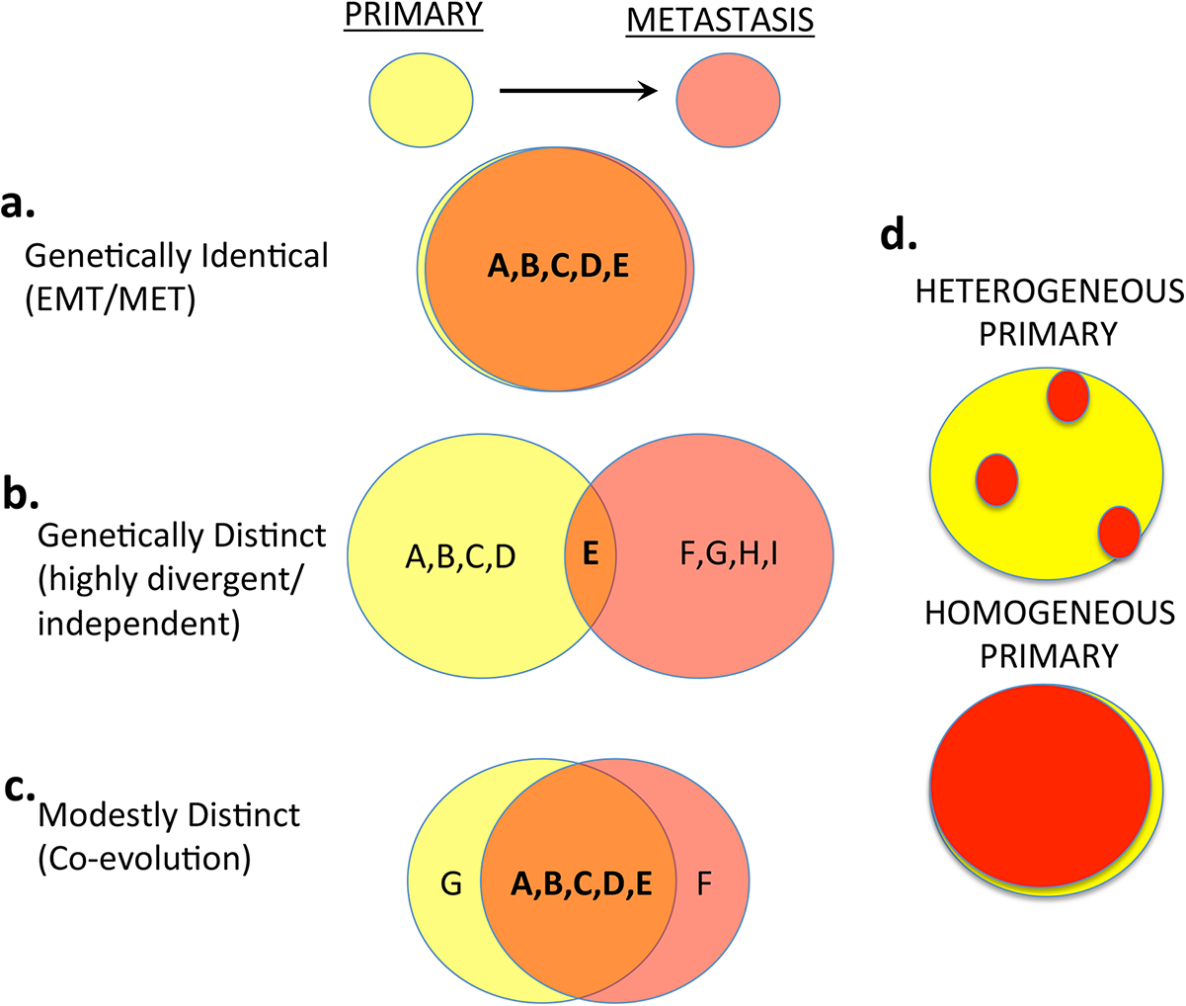
Models for primary and met tumors. **a.** Primary and met are genetically identical, and metastasis occurs via epigenetic or regulatory changes, such as those contributing to EMT/MET phenotypes. **b**. Primary and met are genetically distinct, suggesting the cells diverged rapidly after the split, or that they are independent events. **c.** Primary and met tumors share many mutations, but each has some that are unique. **d.** Illustrations of possible tumor composition.

While the precise mechanisms governing tumor metastasis are still poorly understood, multiple potential explanations have emerged. One notion is that the metastasis is a pure clonal derivative of the primary such that it is nearly genetically identical but for a few new driver genes (Fig 1A) [3]. An extension of this idea is that a tumor might simply undergo a plastic physiological change in gene expression, perhaps unrelated to mutational change, but rather related to environmental clues, resulting in an epithelial to mesenchymal transition (EMT) permitting metastasis [4]. Another notion is that the metastatic lesion is genetically distinct from the primary, due to either the shedding of a highly divergent cell from a heterogeneous primary, or even the origination of a distinct clone (Fig 1B) [5, 6]. A third model suggests primary tumors are genetically similar to metastatic lesions, but not exactly the same. In order to metastasize, the primary tumor must experience additional gain or loss of function via mutation to permit invasion and spread of disease (Fig 1C) [7–9]. Each of these three models is complicated by the possibility of tumor heterogeneity within the primary tumor (Fig 1D).

To gain insight into the sometimes conflicting biological explanations for metastatic behavior, and to better determine which tumor site should be biopsied, we undertook a study of a unique set of tumor samples. In this study, we performed targeted gene sequencing of the 19 tumor pairs using a massively-parallel next-generation sequencing platform on cohorts of paired primary and metastatic CRC tumors.

## Materials and Methods

### Inclusion Criteria

Paired colorectal cancer samples were identified at H. Lee Moffitt Cancer Center as part of a large population based study acquiring nearly 20,000 snap frozen, clinically characterized cancer specimens [10, 11]. Synchronous and metachronous colorectal cancers were all included.

### Tumor Specimen/DNA extraction

Primary and metastatic samples from over 2,000 colorectal cancer patients were available for analysis. In all cases, tissue and clinical data were collected on patients under institutional review board approval as part of the Total Cancer Care (TCC) project [10]. Approval to analyze clinical data from patients whose tumors were used for targeted sequencing was received for this study from the University of South Florida (USF) institutional review board on June 11, 2014, providing a waiver of HIPAA authorization and consent for this retrospective, de-identified study. Additionally, category 4 exemption and waivers were approved by the Spartanburg Regional Institutional Review Board in September 2013, valid until September 2019.

All tumors were collected from curative survival resections and snap frozen in liquid nitrogen within 15–20 min of extirpation. Tumors then underwent a macrodissection quality control process to ensure >80% tumor was present in the specimen that underwent sequence analysis (allowing for sensitive mutation detection). Normal tissue, necrotic tissue and excessive stromal tissues were dissected away from the specimen under frozen section control. DNA was then extracted from 468 CRC specimens, followed by targeted sequencing using a custom designed Agilent Sure Select Capture, Agilent Technologies, Inc., Santa Clara, CA. 1,321 cancer-associated genes were selected by a joint committee (Merck Co., Inc & Moffitt Cancer Center) for hybrid capture and sequencing. Capture probes for the 1,321 genes were based on the Agilent 50MB Sure Select capture (See Table A in S1 File for the list of genes).

### Sequencing and Analysis

#### Variant data

An average of 1.4GB of targeted gene sequencing data (1,321 genes covering 3.8 MB) was generated for each tumor sample using paired-end 90bp sequencing-by-synthesis technology (GAIIx, Illumina, Inc., San Diego, CA) by BGI (Shenzhen, China). The Burrows-Wheeler Aligner (BWA [12]) was used to align sequences to human reference hg19. The Genome Analysis ToolKit (GATK [13]) was used for insertion/deletion realignment, quality score recalibration, and variant identification. ANNOVAR [14] was used to annotate mutations. Although matched normal samples were not available for the 19 tumor-metastatic individuals, we enriched for somatic mutations by removing variants with a global minor allele frequency >1% in the 1000 Genomes project dataset. Additionally, data from 523 normal tissue or blood samples from the TCGA breast dataset were downloaded from dbGAP and analyzed using the same BWA/GATK pipeline as our sequenced samples. A total of 21,179 non-silent variants that were identified in the normal tissues were also used for filtering predicted germline variants, further enriching for somatic mutations. Filtering with the unmatched normal dataset also reduced the number of analytic artifacts (mutations observed frequently in a normal dataset analyzed with the same methods, but not in 1000 Genomes, are likely to be artifacts). Mutation counts are based on filtered data. Mutations in matched samples were directly compared to identify differences. Potential differences were manually reviewed with samtools tview and mpileup [15]. Mutations that appeared to be alignment artifacts based on manual review considering strand imbalance, many mutations in same read, presence of clipping near the mutation position, non-random position of mutation in the reads, low mapping quality, and evidence of the read in normal samples (and not 1000 Genomes) were excluded from the analysis. This step disqualified 12.3% of potential differential mutations, and 26% of these were in *Muc4*, a gene suspected to harbor artifact variants [16, 17]. Samples were considered different at a position if the mutation was present in one sample (generally >10%, our empirically determined allele frequency sensitivity given ~140x average depth of coverage from this and other projects from the same parent dataset of ~4,000 samples), and absent or rarely seen in the other. To avoid false differences resulting from differential mutation determinations, alignments at mutation positions were manually reviewed: This manual review also allowed us to identify false-differences; that is, when allele frequencies at a position were similar, or a mutation was reliably observed in the “reference” sample (despite a differential determination), we reclassified these to be concordant mutations.

## Results

### Clinical data

From 2008–2010, we collected 488 CRC specimens related to 468 sequenced patients to evaluate the genomic profile of CRC from primary or metastatic tissue. Out of these samples, 18 patients had available genomic profiles from more than one tissue specimen (i.e. paired primary:metastatic samples). Fifteen patients had both primary and metastatic/local regional lymph node specimens, two had separate metastatic specimens, and one patient had two primaries and two metastatic tissue specimens available. Therefore a total of 19 paired samples were available. Clinical characteristics are described in Table 1. Paired specimens initially presented with synchronous lesions in 16 instances (84%), while three pairs involved metachronous metastatic disease. Distant metastatic sites included liver (N = 8), lymph nodes (N = 7), lung (N = 3), and ovary (N = 1).

**Table 1:** Clinical Characteristics of the 18 Colorectal Cancer Patients.

Patient	Sex	Age	Primary Tumor site	Metastatic site	Primary Tumor Stage	Synchronous vs metachronous	Stage at time of diagnosis	Survival (months)
A	M	74	Asc. Right	Lymph	T4N2	Synchronous	4	26
B	F	86	Asc. Right	Lymph	T4N2	Synchronous	4	5
C	F	70	Desc. Left	Lymph	T3N1	NA	3	47+
D	F	79	Asc. Right	Lymph	T3N2	NA	3	0
E1	M	64	Asc. Right	Lymph	T3N2	NA	3	31
E2	M	64	Asc. Right	Lymph	T3N2	NA	3	11
F	M	38	Transverse	Lymph	T4N1	NA	3	35+
G	F	67	Rectosig.	Liver	T3N2	Synchronous	4	30+
H	M	66	Rectosig.	Liver	T3N0	Synchronous	4	20+
I	M		Asc. Right	Liver	T3N2	Synchronous	4	22
J	F	81	Asc. Right	Thoracic	T4N2	Synchronous	4	31
K	M	58	Sigmoid	Thoracic	T3N2	Metachronous	3	29+
L	F	60		Thoracic (2)	TxNx	Metachronous	3	32+
M	F	74	Sigmoid	Liver	T3N1	Synchronous	4	3
N	F	44	Sigmoid	Liver	T4N2	Synchronous	4	38
O	M	60	Sigmoid	Liver (2)	TxNx	Metachronous	4	31+
P	F	60	Sigmoid	Ovary	T4N2	Synchronous	4	24+
Q	M	35	Desc. Left	Liver	T4N2	Synchronous	4	5+
R	F	49	Asc. Right	Liver	T3N1	Synchronous	4	13

Note: Asc. = Ascending, Desc. = Descending.

### Mutational profiling and MSI testing

A median of 16,209,684 reads was generated for each sample, with 15,785,209 aligning to the reference human genome, and 5,390,906 aligning within 25 bp of the target regions. Median coverages at targeted bases were: 94.3% > = 10x; 90.7% > = 20x, and 79.6% > = 50x. The median of average depth of coverage across targeted bases was 146.7x (see Table B in S1 File for details on sample_seq_metrics).

In profiling the mutational status of the patients, we specifically focused on well-known genes such as *APC*, *KRAS*, *TP53*, *BRAF* and *PIK3CA*. MSI status was also evaluated as well by genetic analysis of microsatellites in all cases. An *APC* mutation was seen in 16 out of 18 patients and TP53 mutations were seen in 12 patients. *KRAS* mutations were found in 9 patients and located at codons 12, 13, or 61. *PIK3CA* mutations were detected in three patients. Notably, none of the patients had a *BRAF(V600E)* mutation. One patient was MSI-H, 4 patients were MSI-L, but only in one of the tissues, while the remaining 13 patients were MSS.

### Concordance of mutations in matched pairs of primary carcinomas and derivative metastases

**Fig 2:**
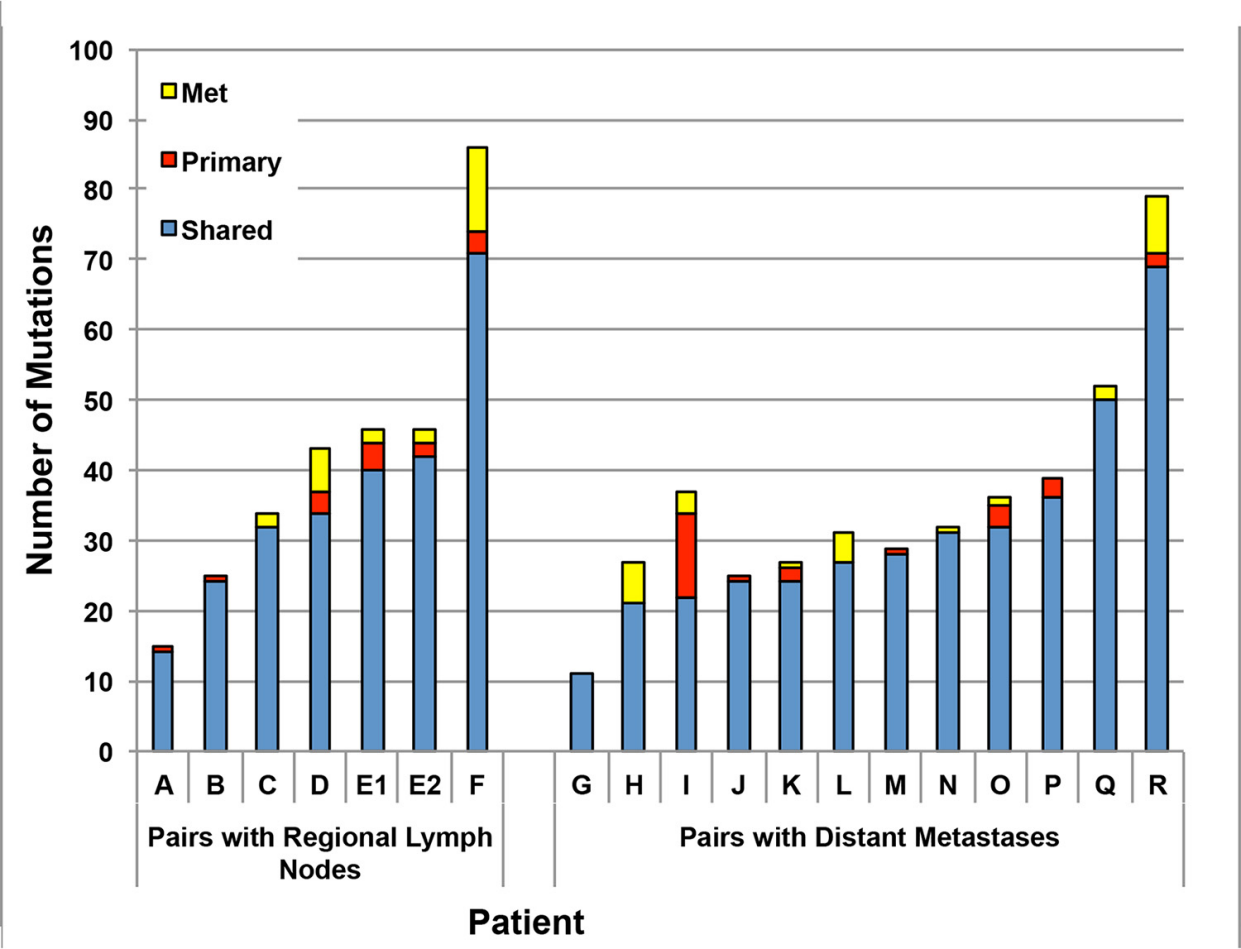
Shared and unique mutations among the paired primary and metastatic samples. The vast majority of mutations are shared. Pairs A through F represent regional lymph node metastases and pairs G through R represent distant metastases.

We examined the mutational concordance of a cohort of 19 pairs of tumors (Fig 2, Table 2, exact positions, depths, and allele frequencies are described in Table C in S1 File). From the 38 tumors, 1,352 putative somatic alterations were identified among amongst 1,321 genes. Of these, 1,264 alterations occurred in both samples (mean = 33.3/sample), 35 occurred only among the 17 primary samples (mean = 2.1), and 53 occurred only in the 21 metastatic samples (mean = 2.5). Notably, the counts were remarkably similar for the primary:lymph node and primary:distant metastasis pairs (mean shared alterations = 36.7 and 31.2). Thus, the overall concordance rate for mutations was 93.5%. However, nearly all (18/19 (94.7%)) paired tumors showed at least one mutational discordance.

**Table 2:** Colon Cancer Patients with Paired Data.

	Pair	Key driver mutations	Shared muts	Discrepant mutations			Site	MSI Status
				Primary	Met	Total		
**Pairs with Regional Lymph Nodes**	A	APC, NRAS	14	1	0	1	Lymph	MSS
	B	APC, TP53	24	1	0	1	Lymph	MSS (M, ND)
	C	KRAS, TP53	32	0	2	2	Lymph	MSS
	D	APC, TP53, PI3K	34	3	6	9	Lymph	MSS
	E1	APC (P), KRAS, TP53	40	4	2	6	Lymph	***MSI-L(P)***
	E2	APC, KRAS, TP53	42	2	2	4	Lymph	MSS
	F	APC, PI3K	71	3	12	15	Lymph	***MSI-H***
	Average (Lymph node pairs)		36.7	2.0	3.4	5.4		
**Pairs with Distant Metastases**	G	APC	11	0	0	0	Liver	MSS (M, ND)
	H	APC (P), KRAS, TP53 (M)	21	0	6	6	Liver	MSS
	I	APC, KRAS, TP53 (M)	22	12	3	15	Liver	MSS
	J	APC, TP53	24	1	0	1	Thoracic	***MSI-L(P)***
	K	APC, TP53	24	2	1	3	Thoracic	MSS
	L	APC, KRAS, TP53, PI3K	27	—	0; 4	4	Thoracic	MSS
	M	APC, KRAS	28	1	0	1	Liver	***MSI-L(M)***
	N	APC, TP53	31	0	1	1	Liver	MSS
	O	APC, TP53	32	—	3; 1	4	Liver	MSS
	P	APC, TP53	36	3	0	3	Ovary	MSS
	Q	APC, KRAS	50	0	2	2	Liver	MSS
	R	KRAS	69	2	8	10	Liver	***MSI-L(M)***
	Average (Distant met pairs)		31.2	2.1	2.1	4.2		
**Average (Overall)**			33.3	2.1	2.5	4.6		

Notes: MSI-H = microsatellite instable – high; MSS microsatellite stable; P = Primary, M = Metastatic,

ND = Not Done.

Mutational differences observed in sample pairs are listed in Table 3. The *TP53(R196X)* alteration was seen in 10 of the other 450 patients who did not have paired samples; we refer to such mutations as *recurrent*. Other differential mutations that were recurrent in our larger cohort were *APC(R223X)* in 7 patients, *APC(E1268X)* in 3 patients, *TP53(E294fs)* (note: other alterations were E294X) in 2 patients, and *JAK1(802_803del)* and *HSP90AB1(549_550del)* occurring in one other patient.

**Table 3:** Discrepant Non-silent Mutations among paired samples from 18 Patients.

**Pair**	**Primary Tissue**	**Regional Lymph node**
A	SMAD4	**—**
B	DGKB	——
C	**——**	MACF1, PTPRC
D	**JAK1 (1),** RASA1, ZNF217	MGMTk, PREX1, RPS6KB2, TCF12, TOP2B, TPO
E1	**APC (7),** ERBB4, MARK1, TTN	STIM1, TRAF4
E2	NTRK2, ZNF831	PTPN13, TRAF4
F	**HSP90AB1 (1),** ABCA3, ITGA10	CIC, EPHA5, ETV4, KIAA1409, MGA, MMP2,PPM1H, PRKCZ, SIRT6, SNX13, TCF3, WNT2
	**Primary Tissue**	**Metastatic Tissue**
G	——	**——**
H	**——**	**APC (3), TP53 (10)**, CASC5, CHD5, FBXW7, KNTC1
I	**TP53 (2)**, BRCA2, CX3CR1, MACF1, MAGI2, PARP14, SMAD2, SMAD3, SYNE1, TNKS, TTN, WNT2	ADCY1, MAST4, NOS1
J	TEX14	——
K	PIK3CG, ROBO1	MAPK10
L	——	1:——2: CSMD3, HERC1, MUC16, PTPRD
M	ITGAL	**——**
N	——	FANCG
O		1: GPC5, PCM1, VRTN2: ADAMTS18
P	ADAMTS20, LRP1B, MAP3K5	——
Q	——	MPL, TACR3
R	TTN, TTN	BCL9, ADAMTS20, COL7A1, RASA1, RB1CC1,ROBO1, TOPBP1, TTN

* Genes in bold are recurrent mutations, with the number in parentheses being the other samples having a mutation in the same position. Genes underlined represent instances in which no alternate reads were identified in the paired tissue lacking the mutation.

Seven genes had two or more discordant alterations (Table 4). They are presented in descending order of their discordant mutation rates, which is the number of mutations per nucleotide base per tissue specimen. Six of them have exactly 2 discordant alterations, so the mutation rate in smaller genes is higher. Five unshared alterations were seen in *TTN*; however, given its size, the unshared mutation rate was lowest among the seven genes. Special attention should be given to the *TP53* and *APC* mutations, as they are known to be driver mutations in colon cancer. Besides the *APC* gene, which occurs in over two-thirds of colorectal cancer, *WNT2* and *MACF1* also impact the Wnt pathway, while *RASA1* is in the RAS pathway. Genes of special interest with a single unshared mutation include *SMAD2*, *SMAD3*, *SMAD4*, and *FBXW7*; additionally, three PTPs (*PTPN13*, *PTPRC*, *PTPRD*) were discordant.

**Table 4:** Genes with Multiple Discrepant Mutations Among the 18 Paired Samples.

Gene	AAs	Recurrent mutations*	Novel mutations*	Rate
WNT2	360	—	R, F	48.7
TP53	393	R; P	—	44.6
RASA1	1047	—	B, I	16.8
ADAMTS20	1910	—	Q, I	9.2
APC	2843	D1, P	—	6.2
MACF1	7788	—	R, N	2.3
TTN	34350	—	D1, R, I(3)	1.3

* Recurrent mutations are mutations that some other CRC patients from the cohort (N = 468) have; novel mutations are unique mutations

Rate = 10^6^ x Mutations/(3 X AAs X 38).

A number of the genes identified as discordant have been found to be mutated in the TCGA CRC dataset at frequencies >10% (e.g. *SMAD4*, *ZNF831*, *ZNF217*, *HSP90AB1*, *TP53*, *SYNE1*, *SMAD2*, *TTN*, *MACF1*, *PREX1*,*FBXW7*, *CSMD3*).

## Discussion

Metastasis is thought to be largely a clonal process whereby the originating cell for the metastatic lesion is derived from the primary lesion, strongly suggesting the two should be closely related [3], although in dispute is precisely how different the primary and metastatic lesions are relative to their mutations, expressed genes and proteins. Also unknown is the relative heterogeneity of the primary tumor. For example, is the tumor largely composed of cells with high metastatic potential and only a few escape due to a stochastic event leading to metastasis, or is it the case that only a few cells in the primary tumor have gained metastatic potential and reside in some unknown geographic location within the primary lesion? The former concept would support the notion that biopsy of the primary lesion is sufficient to predict metastatic potential as well as potential drug targets, whereas the latter biology would result in biopsy sampling errors leading to inappropriate clinical conclusions. The former concept could also lead to the occasional pattern of diffuse and deadly shotgun metastasis vs the usual oligometastatic pattern seen. Another entirely different possibility is that no new mutational events are required for metastasis, but rather it is a physiological event triggered by stromal clues. This theory is embodied in the concept of the epithelial to mesenchymal transition where a plastic transitory mesenchymal state is required for a tumor cell to invade and travel to distant metastatic sites where a reversal of the process (MET) results in a re-capitulation of the primary tumor biology. There are *in vivo* and *in vitro* models of metastasis showing variable gene expression between primary lesions and their cognate metastatic lesions without substantial genomic changes supporting this theory. For example, classic studies have shown high metastatic potential can be acquired via serial “phenotypic” selection or even sorting of cells from an original poorly-metastatic clone. If this is the case, genetics of the primary and metastatic tumor might be nearly identical, permitting biopsy of either lesion.

NGS studies have recently been undertaken to shed light on some of these issues. Recent studies using NGS of other malignancies such as breast and renal cell carcinoma have supported the hypothesis that there are significant genetic differences between the primary lesions when compared to metastatic sites that can permit an evolutionary tracking of development [18, 19]. Interestingly, a recent study in colon cancer showed that remarkably only half of metastatic CRC lesions tested had the same clonal origin with their primary tumor while the rest of the cases were genetically distinct [6]. These sorts of studies might suggest that every metastatic lesion must be biopsied in order to fully understand the complexity of the disease. Other studies, however, are suggesting that metastases may be more like their primary lesions than not [20, 21]. In support of this notion, there is a recent colorectal study showing a high concordance of commonly mutated genes such as *KRAS*, *NRAS*, *BRAF*, *PIK3CA*, and *TP53* between primary and metastatic sites [22]. This is also very similar to the most recently published study in lung cancer where 15 paired non-small cell lung cancers were evaluated [23].

To address these important controversial issues, we examined a new set of paired primary and metastatic CRC lesions using a targeted gene analysis of a robust set of 1,321 cancer associated genes with an ~100X sequencing depth coverage. Our data suggested a very high concordance of detected mutational events between primary and metastatic pairs, whether derived from locoregional lymph nodes or distant metastatic sites. Our data seem to contradict a recent CRC study using whole exome sequencing showing large differences between lesions that may be explained by independent primary events (Fig 1B), or may also be sampling errors or noise secondary to low coverage whole exome sequencing where low frequency events are recorded [6]. Our data in colon cancer showed that the majority of mutations identified were shared between a primary tumor and its cognate paired metastatic lesion, including *KRAS*, *APC*, and *PIK3CA* which are commonly tested mutations in our patients.

Despite the high concordance rate we observed overall, nearly all (18/19 (95%)) paired tumors showed at least one mutational discordance. The concern for the clinician then becomes whether that discordance is clinically significant and worthy of a new biopsy. Is it responsible for the metastatic behavior? Is it a new therapy target, or a possible new mode for resistance to therapy? Or does it simply represent inadequate sampling of a tumor which is inherently heterogeneous? Our seven multiply-discordant mutations included two instances each of *APC* and *TP53*, likely potential drivers of tumor progression and metastatic potential. Additionally, three Wnt pathway (*APC*, *MACF1*, *WNT2*) and one Ras pathway (*RASA1*) genes are among the seven. These findings also suggest lethal metastatic cells may arise as a result of additional of new mutational variants. Identifying the characteristics of the primary cancer that can give rise to lethal metastatic cell clones is of high interest. In our study, *TTN* and *TP53* mutations were found in both the primary and metastatic site but each with new alterations supporting the theory of the gain of function lethal metastatic cell clone.

*TP53* is a mutation which has been studied extensively in colon cancer [24]. It has been reported that an increased incidence of *TP53* mutations is associated with secondary lesions of colorectal tumors, suggesting that mutations in this gene may play an important role in the establishment of colorectal liver metastases [25]. For example, p53, the protein product of *TP53*, has been reported to inhibit the Warburg effect and promote mitochondrial oxidative metabolism, an anti-metastasis mechanism, and thus inactivation of this tumor suppressor function contributes to process of metastasis [26]. In our study, 13/19 (68%) pairs had *TP53* mutations, a high rate which is similar to other studies [27]. Interestingly, in our study, one patient had a discordant *TP53* mutation in the metastatic site, while another had one in the primary tumor. The discordant mutations from the paired samples are informative regarding the evolution of the tumor. These findings suggest that *TP53* mutations are frequently a late carcinogenic event, consistent with an evolutionary model set forth by Vogelstein et al [28].

Additional interesting mutations found in metastatic sites were members of the protein tyrosine phosphatase (PTP) family. There were 3 new mutations seen in metastatic lesions. These PTPs have been described as either tumor suppressors or as candidate oncoproteins [29]. Alterations in these genes can result in changes to the equilibrium of kinase–phosphatase activity that might have deleterious effects which could ultimately lead to cancer progression.

Our study notably identified gain of new mutations in both the primary relative to its cognate metastatic paired lesion as well as new mutations in the metastatic lesions relative to the primaries. This observation can be explained by the co-evolution of both the primary and metastatic lesions over time (Fig 1C). It is also possible that these discordant mutations represent different populations of a heterogeneous primary tumor.

There are several limitations to our study. Our small sized cohort included a heterogeneous population, with both synchronous and metachronous tumors. These groups may be inherently different genomically. For example, the new mutations seen in the metastatic sites can be the result of the metastatic process or due to selection of resistant clones induced by previous systemic treatment such as neoadjuvant therapy prior to liver resection. Unfortunately, we do not have clinical chemotherapy data for the patients who developed metachronous disease. Therefore it is unclear what effect if any, chemotherapy or targeted therapies would have on the emergence of new mutations in the metastatic sites. Also intratumoral heterogeneity, while likely not as high in CRC lesions as in geographically distinct renal cell nodules derived from the same primary tumor [28], can be a confounding factor which could explain some of genetic divergence as well. Our study utilized surgically resected specimens thus allowing us to capture a large portion of each tumor and effectively assess its heterogeneity by observing low frequency, sub-clonal variants.

## Conclusions

Our study showed a high level of concordance for potential somatic alterations, suggesting that genomic profile of primary site is very similar to that of the metastatic site for the majority of interrogated cancer genes. Thus, biopsy of the primary tumor may be sufficient in chemo-naïve patients. However, we also believe there are cases where new biopsies are likely needed to accurately assess the mutational landscape of a tumor and design appropriate therapy, for example, in CRC patients who progress after certain therapy (i.e. anti EGFR therapy). In this situation, it is possible that a resistant subclone has emerged under selective pressure of the targeted therapy. There are also occasions where response will only be seen in the primary tumor and not in the metastatic site. In this scenario, it is possible that new mutations have occurred in the metastatic site leading to resistance. These data would justify the need for a new biopsy of the metastatic site to better understand the acquired resistance or new actionable mutations so that patients could be offered personalized treatment. Therefore it may be important that future studies allow for new biopsies of metastatic sites to better understand metastasis and acquired resistance.

## Supporting Information

S1 File Combined file of supporting tables. Table A. The list of 1321 genes for targeted sequencing; Table B. The sample sequencing metrics; Table C. The Mutation sequencing metrics.
(DOCX)
