# Supplementary material for: Co-Evolution of Somatic Variation in Primary and Metastatic Colorectal Cancer May Expand Biopsy Indications in the Molecular Era
Source: PLoS One. 2015 May 14;10(5):e0126670. doi: 10.1371/journal.pone.0126670 (PMC4431733; doi:10.1371/journal.pone.0126670)
Supplement: S1 File — Table A. The list of 1321 genes for targeted sequencing; Table B. The sample sequencing metrics; Table C. The Mutation sequencing metrics. (DOCX) [file pone.0126670.s001.docx]

**Supporting Information**

**Table A, Related to Experimental Procedures. List of 1321 genes in targeted gene sequencing**

| *ABCA1* | *ABCA3* | *ABCB1* | *ABCC2* | *ABCC3* | *ABCC4* | *ABL1* | *ABL2* |
| --- | --- | --- | --- | --- | --- | --- | --- |
| *ACAP1* | *ACVR1B* | *ACVR2A* | *ADA* | *ADAM17* | *ADAM29* | *ADAM33* | *ADAMTS18* |
| *ADAMTS20* | *ADAMTSL3* | *ADCY1* | *ADCY9* | *ADH7* | *ADHFE1* | *ADORA2A* | *ADORA2B* |
| *ADRA1A* | *ADRB3* | *AFF4* | *AKAP6* | *AKAP9* | *AKT1* | *AKT1S1* | *AKT2* |
| *AKT3* | *ALK* | *ALOX12B* | *ALOX15* | *ALPK2* | *ALPK3* | *ALS2* | *ANAPC5* |
| *ANKRD32* | *ANO1* | *APAF1* | *APBB1IP* | *APC* | *APC2* | *APEX1* | *APOB* |
| *AR* | *ARAF* | *ARAP3* | *AREG* | *ARFRP1* | *ARHGAP26* | *ARHGAP29* | *ARHGAP6* |
| *ARHGEF11* | *ARHGEF12* | *ARID1A* | *ARID3A* | *ARNT* | *ARRB1* | *ASXL1* | *ATG10* |
| *ATG12* | *ATG16L1* | *ATG16L2* | *ATG2B* | *ATG4D* | *ATG5* | *ATG9B* | *ATM* |
| *ATOH1* | *ATP8B1* | *ATR* | *ATRX* | *AURKA* | *AURKB* | *AURKC* | *AXIN1* |
| *AXIN2* | *AXL* | *AZI2* | *BAD* | *BAI1* | *BAI2* | *BAI3* | *BAK1* |
| *BAP1* | *BARD1* | *BAT1* | *BAX* | *BAZ1A* | *BBC3* | *BCAT1* | *BCL10* |
| *BCL11A* | *BCL2* | *BCL2A1* | *BCL2L1* | *BCL2L11* | *BCL2L14* | *BCL2L15* | *BCL2L2* |
| *BCL3* | *BCL6* | *BCL9* | *BCL9L* | *BCORL1* | *BCR* | *BECN1* | *BID* |
| *BIK* | *BIRC2* | *BIRC3* | *BIRC5* | *BIRC6* | *BIRC7* | *BLM* | *BMF* |
| *BMI1* | *BMP2* | *BMP4* | *BMPR1A* | *BMPR1B* | *BMPR2* | *BMX* | *BNIP3* |
| *BOK* | *BPTF* | *BRAF* | *BRCA1* | *BRCA2* | *BRD2* | *BRD3* | *BRIP1* |
| *BTG2* | *BTK* | *BTRC* | *BUB1* | *C10orf137* | *C12orf63* | *C1orf144* | *C9orf96* |
| *CA12* | *CA9* | *CABLES1* | *CAD* | *CALCR* | *CALCRL* | *CARD11* | *CASC5* |
| *CASK* | *CASP2* | *CASP8* | *CASP9* | *CAV1* | *CBFA2T3* | *CBL* | *CBLB* |
| *CBLC* | *CBX4* | *CBX7* | *CBX8* | *CCBP2* | *CCDC63* | *CCDC82* | *CCKAR* |
| *CCKBR* | *CCL18* | *CCL2* | *CCL8* | *CCNA1* | *CCNA2* | *CCND1* | *CCND2* |
| *CCND3* | *CCNDBP1* | *CCNE1* | *CCNE2* | *CCR3* | *CCR5* | *CD14* | *CD22* |
| *CD248* | *CD33* | *CD34* | *CD4* | *CD44* | *CD79A* | *CD79B* | *CD8A* |
| *CDC25A* | *CDC25B* | *CDC25C* | *CDC42* | *CDC42BPA* | *CDC42BPB* | *CDC6* | *CDC7* |
| *CDC73* | *CDH1* | *CDH11* | *CDH2* | *CDH20* | *CDH5* | *CDK1* | *CDK12* |
| *CDK19* | *CDK2* | *CDK4* | *CDK5* | *CDK6* | *CDK7* | *CDK8* | *CDK9* |
| *CDKL2* | *CDKN1A* | *CDKN1B* | *CDKN1C* | *CDKN2A* | *CDKN2B* | *CDKN2C* | *CDKN2D* |
| *CDKN3* | *CDS1* | *CEBPA* | *CELSR1* | *CELSR2* | *CENPF* | *CEP110* | *CES3* |
| *CHD5* | *CHD8* | *CHEK1* | *CHEK2* | *CHIC2* | *CHKA* | *CHKB* | *CHRNA3* |
| *CHRNA5* | *CHRNB4* | *CHUK* | *CIC* | *CLSPN* | *CLTC* | *CLU* | *CMAS* |
| *CNTD2* | *CNTN1* | *COL11A1* | *COL14A1* | *COL1A1* | *COL7A1* | *CREBBP* | *CRHR1* |
| *CRKL* | *CRLF2* | *CSDA* | *CSF1R* | *CSK* | *CSMD3* | *CSNK1G2* | *CTDP1* |
| *CTNNA1* | *CTNNB1* | *CTNND2* | *CTSH* | *CUBN* | *CX3CR1* | *CXCR4* | *CXorf30* |
| *CYB5D2* | *CYLD* | *CYP1B1* | *CYP2C19* | *CYP2C8* | *CYP2D6* | *CYP3A4* | *CYP3A5* |
| *DAB2* | *DAXX* | *DBF4* | *DBF4B* | *DBN1* | *DCC* | *DCLK3* | *DCLRE1C* |
| *DDB1* | *DDB2* | *DDIT3* | *DDR1* | *DDR2* | *DDX1* | *DDX10* | *DDX11* |
| *DGKA* | *DGKB* | *DGKG* | *DGKZ* | *DIP2C* | *DIS3* | *DKK1* | *DLC1* |
| *DLG1* | *DLL1* | *DLL3* | *DLL4* | *DNAH8* | *DNAJC24* | *DNMT1* | *DNMT3A* |
| *DNMT3B* | *DNTT* | *DOK1* | *DOT1L* | *DPYSL4* | *DTX1* | *DUSP1* | *DUSP10* |
| *DUSP14* | *DUSP2* | *DUSP4* | *DUSP5* | *DUSP6* | *DUSP9* | *DYRK2* | *E2F1* |
| *E2F3* | *E4F1* | *ECT2* | *EEF2K* | *EGF* | *EGFL6* | *EGFR* | *EGLN1* |
| *EIF2AK1* | *EIF4E* | *ELK1* | *ELK3* | *ELP2* | *ENPP2* | *EP300* | *EP400* |
| *EPHA1* | *EPHA10* | *EPHA2* | *EPHA3* | *EPHA4* | *EPHA5* | *EPHA6* | *EPHA7* |
| *EPHA8* | *EPHB1* | *EPHB2* | *EPHB3* | *EPHB4* | *EPHB6* | *EPO* | *ERBB2* |
| *ERBB3* | *ERBB4* | *ERC1* | *ERCC2* | *ERCC3* | *ERCC4* | *ERCC5* | *ERCC6* |
| *EREG* | *ERG* | *ERGIC3* | *ERN1* | *ERN2* | *ERRFI1* | *ESR1* | *ESR2* |
| *ETS1* | *ETS2* | *ETV1* | *ETV4* | *ETV5* | *ETV6* | *EVC2* | *EWSR1* |
| *EXOC2* | *EXOC4* | *EXT1* | *EXT2* | *EZH2* | *F2RL2* | *FADD* | *FAM123B* |
| *FAM46A* | *FANCA* | *FANCB* | *FANCC* | *FANCD2* | *FANCE* | *FANCF* | *FANCG* |
| *FANCL* | *FANCM* | *FAS* | *FASLG* | *FASN* | *FBXL6* | *FBXO10* | *FBXO32* |
| *FBXW7* | *FCGR3A* | *FEN1* | *FER* | *FES* | *FGF10* | *FGF19* | *FGF2* |
| *FGF3* | *FGF4* | *FGFR1* | *FGFR2* | *FGFR3* | *FGFR4* | *FH* | *FHIT* |
| *FIGF* | *FKTN* | *FLI1* | *FLNB* | *FLT1* | *FLT3* | *FLT4* | *FN1* |
| *FOS* | *FOSL1* | *FOXL2* | *FOXM1* | *FOXO1* | *FOXO3* | *FOXP3* | *FOXP4* |
| *FPR3* | *FRMD7* | *FSCB* | *FSCN1* | *FURIN* | *FUT4* | *FYN* | *FZD10* |
| *G3BP2* | *GAB1* | *GAB2* | *GAB3* | *GABARAPL1* | *GABPA* | *GADD45G* | *GATA1* |
| *GEN1* | *GLI1* | *GLI3* | *GLP1R* | *GLS* | *GNA11* | *GNAQ* | *GNAS* |
| *GOLIM4* | *GPC5* | *GPR124* | *GPR81* | *GPR84* | *GRB10* | *GRB2* | *GRIN2A* |
| *GRK5* | *GRM1* | *GRM3* | *GRM5* | *GRM6* | *GRM7* | *GRM8* | *GSK3A* |
| *GSK3B* | *GSPT1* | *GSTP1* | *GSTT1* | *GSX2* | *GTPBP4* | *GUCY1A2* | *GUCY2F* |
| *HAPLN1* | *HCCS* | *HCRTR2* | *HDAC1* | *HDAC2* | *HDAC4* | *HDAC9* | *HDLBP* |
| *HECW1* | *HECW2* | *HERC1* | *HERC3* | *HERC6* | *HES1* | *HES4* | *HES5* |
| *HES6* | *HES7* | *HEY1* | *HEY2* | *HEYL* | *HGF* | *HIF1A* | *HIP1* |
| *HIPK2* | *HLA-A* | *HMGA1* | *HMGA2* | *HNF1A* | *HOXA3* | *HOXA9* | *HOXD13* |
| *HRAS* | *HRK* | *HSD11B1* | *HSD17B2* | *HSF1* | *HSP90AA1* | *HSP90AB1* | *HSPA2* |
| *HUS1* | *ICK* | *ID1* | *IDH1* | *IDH2* | *IGF1* | *IGF1R* | *IGF2* |
| *IGF2R* | *IGFBP1* | *IGFBP2* | *IGFBP3* | *IGFBP4* | *IGFBP5* | *IGFBP6* | *IGFBP7* |
| *IGFBPL1* | *IKBIP* | *IKBKAP* | *IKBKB* | *IKBKE* | *IKBKG* | *IKZF1* | *IKZF3* |
| *IL12A* | *IL12B* | *IL1B* | *IL21R* | *ILF3* | *ILK* | *ING4* | *INHBA* |
| *INPP4A* | *INPP4B* | *INPP5A* | *INPPL1* | *INSR* | *INSRR* | *IQGAP1* | *IRAK2* |
| *IRAK3* | *IRAK4* | *IRF4* | *IRS1* | *IRS2* | *IRS4* | *ITCH* | *ITGA10* |
| *ITGA9* | *ITGAL* | *ITGAV* | *ITGB2* | *ITGB3* | *ITGB4* | *ITK* | *ITPA* |
| *ITPR1* | *ITPR2* | *ITPR3* | *ITSN2* | *JAG1* | *JAG2* | *JAK1* | *JAK2* |
| *JAK3* | *JUN* | *JUNB* | *KAT2B* | *KCNH8* | *KDM1A* | *KDM4C* | *KDM5A* |
| *KDM5C* | *KDM6A* | *KDR* | *KEAP1* | *KIAA0182* | *KIAA1409* | *KIF16B* | *KISS1* |
| *KIT* | *KLF6* | *KNTC1* | *KRAS* | *KSR1* | *KSR2* | *KTN1* | *LAMC1* |
| *LAMP1* | *LARGE* | *LATS1* | *LATS2* | *LCK* | *LCP1* | *LDHA* | *LDHB* |
| *LEF1* | *LGALS7* | *LGR5* | *LHCGR* | *LIG3* | *LIG4* | *LMO2* | *LPHN1* |
| *LPHN2* | *LPHN3* | *LRP1* | *LRP1B* | *LRP5* | *LRP6* | *LRRC10* | *LRRC7* |
| *LRRK2* | *LRRN3* | *LTA* | *LTBP1* | *LTBR* | *LTF* | *LTK* | *LYN* |
| *MACF1* | *MAG* | *MAGI1* | *MAGI2* | *MAGI3* | *MAMDC4* | *MAN1B1* | *MAP1LC3A* |
| *MAP1LC3B* | *MAP1LC3C* | *MAP2K1* | *MAP2K2* | *MAP2K4* | *MAP2K6* | *MAP2K7* | *MAP3K1* |
| *MAP3K11* | *MAP3K14* | *MAP3K2* | *MAP3K5* | *MAP3K6* | *MAP3K8* | *MAP3K9* | *MAP4K4* |
| *MAPK1* | *MAPK10* | *MAPK13* | *MAPK3* | *MAPK7* | *MAPK8* | *MAPK8IP2* | *MAPK8IP3* |
| *MAPK9* | *MAPKAP1* | *MAPT* | *MARK1* | *MARK4* | *MAS1* | *MAS1L* | *MAST4* |
| *MCHR2* | *MCL1* | *MCM3AP* | *MCPH1* | *MDC1* | *MDK* | *MDM2* | *MDM4* |
| *MECOM* | *MEF2C* | *MELK* | *MEN1* | *MERTK* | *MET* | *MEX3B* | *MGA* |
| *MGC42105* | *MGMT* | *MICAL1* | *MINK1* | *MITF* | *MKNK1* | *MKNK2* | *MKRN3* |
| *MLH1* | *MLH3* | *MLL* | *MLL2* | *MLL3* | *MLL4* | *MLLT6* | *MMP16* |
| *MMP2* | *MMP8* | *MMP9* | *MN1* | *MOS* | *MPL* | *MRE11A* | *MS4A1* |
| *MSH2* | *MSH3* | *MSH4* | *MSH6* | *MSN* | *MST1R* | *MTHFR* | *MTM1* |
| *MTMR3* | *MTOR* | *MUC1* | *MUC16* | *MUC4* | *MUTYH* | *MYB* | *MYBL1* |
| *MYBL2* | *MYC* | *MYCL1* | *MYCN* | *MYD88* | *MYEOV* | *MYH1* | *MYH11* |
| *MYH9* | *MYLK2* | *MYO1B* | *MYST4* | *N4BP2* | *NBN* | *NCAM1* | *NCDN* |
| *NCK1* | *NCK2* | *NCOA2* | *NCOA7* | *NEDD4L* | *NEIL3* | *NEK10* | *NEK11* |
| *NEK2* | *NEK7* | *NEK8* | *NEK9* | *NF1* | *NF2* | *NFATC3* | *NFKB1* |
| *NFKB2* | *NFKBIB* | *NFKBID* | *NFKBIE* | *NFKBIZ* | *NGFR* | *NIN* | *NIPBL* |
| *NKD2* | *NKX2-1* | *NLE1* | *NLK* | *NLRP1* | *NLRP3* | *NLRP5* | *NLRP8* |
| *NMUR2* | *NOS1* | *NOS2* | *NOTCH1* | *NOTCH2* | *NOTCH3* | *NOTCH4* | *NOV* |
| *NPM1* | *NPY2R* | *NPY5R* | *NQO1* | *NR0B1* | *NRARP* | *NRAS* | *NRBP1* |
| *NRG2* | *NRK* | *NRP2* | *NTRK1* | *NTRK2* | *NTRK3* | *NUAK1* | *NUAK2* |
| *NUMB* | *NUMBL* | *NUP153* | *NUP214* | *NUP98* | *OBSCN* | *ODZ1* | *OR8G1* |
| *ORAI1* | *ORAOV1* | *ORM1* | *P2RY10* | *P2RY8* | *PAG1* | *PAK1* | *PAK3* |
| *PAK4* | *PAK6* | *PAK7* | *PALB2* | *PARK2* | *PARP1* | *PARP14* | *PARP2* |
| *PARP4* | *PAX3* | *PAX5* | *PAX6* | *PAX7* | *PAXIP1* | *PCDHB2* | *PCGF2* |
| *PCM1* | *PCNA* | *PDCD4* | *PDGFA* | *PDGFB* | *PDGFRA* | *PDGFRB* | *PDGFRL* |
| *PDIA4* | *PDK1* | *PDK3* | *PDPK1* | *PDZRN4* | *PEA15* | *PER1* | *PERP* |
| *PES1* | *PFKFB1* | *PFKFB2* | *PFKFB3* | *PFKFB4* | *PGR* | *PHB* | *PHF14* |
| *PHF20* | *PHLPP1* | *PHLPP2* | *PHOX2B* | *PIAS1* | *PIK3C2A* | *PIK3C2B* | *PIK3C2G* |
| *PIK3C3* | *PIK3CA* | *PIK3CB* | *PIK3CD* | *PIK3CG* | *PIK3R1* | *PIK3R2* | *PIK3R3* |
| *PIM1* | *PIM2* | *PKHD1* | *PKM2* | *PKMYT1* | *PKN3* | *PLA2G4A* | *PLAG1* |
| *PLAGL1* | *PLAT* | *PLAU* | *PLCB1* | *PLCB4* | *PLCG1* | *PLCG2* | *PLCH1* |
| *PLD1* | *PLD2* | *PLXNB3* | *PMAIP1* | *PML* | *PMS2* | *POLE* | *POLL* |
| *POLM* | *POLN* | *POLQ* | *POU1F1* | *PPARA* | *PPARG* | *PPARGC1A* | *PPM1D* |
| *PPM1H* | *PPM1L* | *PPP1CC* | *PPP1R3A* | *PPP2R1A* | *PPP2R2B* | *PPP2R3A* | *PRDM16* |
| *PRDM5* | *PREX1* | *PREX2* | *PRKAA1* | *PRKAA2* | *PRKAB1* | *PRKAB2* | *PRKACA* |
| *PRKACB* | *PRKACG* | *PRKAG1* | *PRKAG2* | *PRKAG3* | *PRKAR1A* | *PRKCA* | *PRKCD* |
| *PRKCE* | *PRKCG* | *PRKCH* | *PRKCI* | *PRKCZ* | *PRKD1* | *PRKD2* | *PRKDC* |
| *PRRC2C* | *PSEN1* | *PSEN2* | *PTCH1* | *PTCH2* | *PTEN* | *PTGFR* | *PTGS2* |
| *PTK2* | *PTK2B* | *PTP4A3* | *PTPN1* | *PTPN11* | *PTPN12* | *PTPN13* | *PTPN14* |
| *PTPN21* | *PTPN22* | *PTPN3* | *PTPN5* | *PTPN6* | *PTPN9* | *PTPRC* | *PTPRD* |
| *PTPRF* | *PTPRG* | *PTPRJ* | *PTPRS* | *PTPRT* | *PTPRU* | *PXN* | *PYGO2* |
| *RAC1* | *RAD18* | *RAD21* | *RAD50* | *RAD51* | *RAD51C* | *RAD54B* | *RAF1* |
| *RALA* | *RALB* | *RALGAPA1* | *RAP1A* | *RAP1GDS1* | *RAPGEF2* | *RAPH1* | *RARA* |
| *RASA1* | *RASA2* | *RASA3* | *RASGRF1* | *RASGRF2* | *RASGRP1* | *RASSF1* | *RB1* |
| *RB1CC1* | *RBBP4* | *RBL1* | *RBL2* | *RBP2* | *RBPJ* | *RECQL4* | *REG4* |
| *REL* | *RELA* | *RELB* | *RET* | *REV1* | *REV3L* | *RFC1* | *RFC4* |
| *RFC5* | *RFX2* | *RGL1* | *RGL2* | *RHEB* | *RHOBTB2* | *RICTOR* | *RIF1* |
| *RING1* | *RIPK1* | *RIPK4* | *RNF123* | *RNF213* | *ROBO1* | *ROBO2* | *ROCK1* |
| *ROCK2* | *ROR1* | *ROR2* | *ROS1* | *RPL10* | *RPL36A* | *RPS6* | *RPS6KA1* |
| *RPS6KA2* | *RPS6KA3* | *RPS6KA4* | *RPS6KA5* | *RPS6KA6* | *RPS6KB1* | *RPS6KB2* | *RPTOR* |
| *RRAS2* | *RRM2B* | *RUNX1* | *RUNX1T1* | *RYK* | *SDHB* | *SDHC* | *SDHD* |
| *SENP6* | *SERPINB5* | *SESN2* | *SETD2* | *SFN* | *SFRP1* | *SFRP2* | *SGK1* |
| *SGK2* | *SGK3* | *SGK494* | *SH3GLB1* | *SHC1* | *SIK1* | *SIRT1* | *SIRT6* |
| *SIX4* | *SKP2* | *SLC17A5* | *SLC19A1* | *SLC22A2* | *SLC2A1* | *SLC2A3* | *SLC2A4* |
| *SLC4A4* | *SLC6A18* | *SLC6A2* | *SLCO1B3* | *SMAD2* | *SMAD3* | *SMAD4* | *SMAD7* |
| *SMARCA4* | *SMARCB1* | *SMC6* | *SMG1* | *SMO* | *SMYD2* | *SNAI1* | *SNAI2* |
| *SNAI3* | *SNX13* | *SOCS1* | *SOD2* | *SORL1* | *SOS1* | *SOS2* | *SOX10* |
| *SOX11* | *SOX2* | *SPEG* | *SPEN* | *SPO11* | *SPOP* | *SPRED1* | *SPRY1* |
| *SPRY2* | *SPTAN1* | *SRC* | *SRF* | *SRPK2* | *SRSF6* | *STAT1* | *STAT3* |
| *STAT4* | *STAT5A* | *STAT5B* | *STIL* | *STIM1* | *STK11* | *STK19* | *STK32B* |
| *STK32C* | *STK33* | *STK36* | *STMN1* | *STMN3* | *STON2* | *STYK1* | *SUFU* |
| *SULT1A1* | *SUZ12* | *SYK* | *SYNE1* | *SYNE2* | *TAB3* | *TACR3* | *TAF15* |
| *TAF1L* | *TAL1* | *TANK* | *TBCK* | *TBK1* | *TBKBP1* | *TBX22* | *TCF12* |
| *TCF3* | *TCF4* | *TCF7L2* | *TDG* | *TEC* | *TECTA* | *TEK* | *TERT* |
| *TET2* | *TEX14* | *TFDP1* | *TFE3* | *TFEB* | *TGFB1* | *TGFB2* | *TGFB3* |
| *TGFBR1* | *TGFBR2* | *THBS1* | *THEM4* | *THRAP3* | *TIAM1* | *TICAM1* | *TICAM2* |
| *TK1* | *TLE4* | *TLN1* | *TLR2* | *TLR3* | *TLR4* | *TLR7* | *TMEFF2* |
| *TMEM132B* | *TMEM161A* | *TMPRSS2* | *TMPRSS6* | *TNF* | *TNFAIP3* | *TNFRSF10A* | *TNFRSF10B* |
| *TNFRSF11A* | *TNFRSF1A* | *TNFRSF8* | *TNFSF11* | *TNK2* | *TNKS* | *TNKS2* | *TNNI3K* |
| *TNPO1* | *TNPO3* | *TOP1* | *TOP2A* | *TOP2B* | *TOPBP1* | *TP53* | *TP53AIP1* |
| *TP53BP1* | *TP63* | *TP73* | *TPD52* | *TPMT* | *TPO* | *TRADD* | *TRAF1* |
| *TRAF2* | *TRAF3* | *TRAF4* | *TRAF5* | *TRAF6* | *TRAF7* | *TRIB3* | *TRIM24* |
| *TRIM28* | *TRIM33* | *TRIM36* | *TRIM37* | *TRIM47* | *TRIO* | *TRIP11* | *TRRAP* |
| *TSC1* | *TSC2* | *TSHR* | *TSPAN31* | *TTBK2* | *TTK* | *TTN* | *TUBD1* |
| *TWF2* | *TWIST1* | *TYK2* | *UBA1* | *UBASH3B* | *UBP1* | *UBR4* | *UBR5* |
| *UGT1A1* | *UHRF1BP1L* | *ULK1* | *ULK2* | *ULK3* | *UMPS* | *UPP1* | *USP24* |
| *USP28* | *USP33* | *USP34* | *USP42* | *USP43* | *USP5* | *USP54* | *USP6NL* |
| *USP7* | *USP8* | *USP9X* | *UVRAG* | *VAV1* | *VEGFA* | *VEGFC* | *VEPH1* |
| *VHL* | *VPS13B* | *VRTN* | *WEE1* | *WHSC1* | *WIF1* | *WNK1* | *WNK2* |
| *WNK4* | *WNT1* | *WNT10B* | *WNT2* | *WNT2B* | *WNT4* | *WNT9B* | *WRN* |
| *WSB1* | *WT1* | *WWP2* | *XBP1* | *XIAP* | *XPA* | *XPC* | *XRCC1* |
| *XRCC2* | *XRCC3* | *XRCC5* | *XRCC6* | *YWHAQ* | *ZAP70* | *ZBTB16* | *ZC3H12B* |
| *ZEB1* | *ZEB2* | *ZMYM2* | *ZMYM4* | *ZNF148* | *ZNF217* | *ZNF384* | *ZNF442* |
| *ZNF831* |  |  |  |  |  |  |  |

**Table B. Sample sequencing metrics**

| **#sample** | **total_reads** | **aligned_reads** | **reads_near_**  **target** | **depth_ge10** | **depth_ge20** | **depth_ge50** | **targeted_**  **positions** | **fraction_**  **target_ge10** | **fraction_**  **target_ge20** | **fraction_**  **target_ge50** | **avg_cov** |
| --- | --- | --- | --- | --- | --- | --- | --- | --- | --- | --- | --- |
| DS-48725 | 17793206 | 17545312 | 6805291 | 3591039 | 3466566 | 3104632 | 3792814 | 0.9468 | 0.914 | 0.8186 | 162.6206 |
| DS-48634 | 18084586 | 17012581 | 10191176 | 3563362 | 3417987 | 2968009 | 3792814 | 0.9395 | 0.9012 | 0.7825 | 145.9299 |
| DS-53055 | 12517172 | 12360475 | 4450809 | 3540802 | 3360429 | 2722973 | 3792814 | 0.9336 | 0.886 | 0.7179 | 104.4509 |
| DS-54174 | 16248702 | 16030673 | 4088574 | 3576873 | 3440106 | 3026996 | 3792814 | 0.9431 | 0.907 | 0.7981 | 155.1447 |
| DS-48595 | 18213916 | 17986418 | 6814745 | 3580458 | 3445434 | 3033845 | 3792814 | 0.944 | 0.9084 | 0.7999 | 158.7403 |
| DS-59982 | 13399676 | 12970468 | 3494367 | 3533382 | 3371701 | 2875163 | 3792814 | 0.9316 | 0.889 | 0.7581 | 116.1136 |
| DS-51202 | 16071848 | 15814663 | 7085641 | 3543216 | 3397598 | 2963294 | 3792814 | 0.9342 | 0.8958 | 0.7813 | 130.7064 |
| DS-51211 | 13837056 | 13539961 | 3580245 | 3567070 | 3410113 | 2910107 | 3792814 | 0.9405 | 0.8991 | 0.7673 | 131.4291 |
| DS-59560 | 17093626 | 15721556 | 5903902 | 3541987 | 3387640 | 2935916 | 3792814 | 0.9339 | 0.8932 | 0.7741 | 146.3444 |
| DS-57659 | 19547514 | 19057348 | 13866925 | 3577985 | 3439735 | 3045755 | 3792814 | 0.9434 | 0.9069 | 0.803 | 147.0195 |
| DS-44604 | 2056034 | 2011885 | 468781 | 2484156 | 1562423 | 333869 | 3792814 | 0.655 | 0.4119 | 0.088 | 20.8864 |
| DS-67176 | 14655330 | 14172536 | 3896777 | 3475264 | 3259328 | 2690212 | 3792814 | 0.9163 | 0.8593 | 0.7093 | 118.336 |
| DS-48789 | 17269994 | 16668374 | 9674598 | 3575079 | 3442509 | 3045865 | 3792814 | 0.9426 | 0.9076 | 0.8031 | 145.9396 |
| DS-54095 | 17172868 | 16833802 | 4488149 | 3590412 | 3466522 | 3109103 | 3792814 | 0.9466 | 0.914 | 0.8197 | 164.8928 |
| DS-50851 | 14497884 | 14140873 | 5018885 | 3510793 | 3298162 | 2723564 | 3792814 | 0.9256 | 0.8696 | 0.7181 | 135.5356 |
| DS-59918 | 15101188 | 14598340 | 3950153 | 3546246 | 3347483 | 2788274 | 3792814 | 0.935 | 0.8826 | 0.7351 | 147.0781 |
| DS-69689 | 18268214 | 17965570 | 13595276 | 3586765 | 3462832 | 3131814 | 3792814 | 0.9457 | 0.913 | 0.8257 | 172.3758 |
| DS-68010 | 20495258 | 20153670 | 5194864 | 3641323 | 3542950 | 3254908 | 3792814 | 0.9601 | 0.9341 | 0.8582 | 210.1646 |
| DS-55324 | 13143154 | 12844722 | 3949299 | 3553966 | 3401441 | 2916883 | 3792814 | 0.937 | 0.8968 | 0.7691 | 117.8375 |
| DS-55353 | 20702712 | 20171026 | 9003364 | 3608132 | 3488618 | 3118458 | 3792814 | 0.9513 | 0.9198 | 0.8222 | 180.2923 |
| DS-52992 | 16769602 | 16475601 | 5603069 | 3588069 | 3451223 | 3020284 | 3792814 | 0.946 | 0.9099 | 0.7963 | 149.3562 |
| DS-54373 | 13209822 | 12952669 | 3363947 | 3561476 | 3407706 | 2957806 | 3792814 | 0.939 | 0.8985 | 0.7798 | 129.0514 |
| DS-54072 | 17026618 | 16723025 | 7101104 | 3600178 | 3472802 | 3097816 | 3792814 | 0.9492 | 0.9156 | 0.8168 | 167.8089 |
| DS-53085 | 14733554 | 14368875 | 6323724 | 3453668 | 3238583 | 2692024 | 3792814 | 0.9106 | 0.8539 | 0.7098 | 134.7425 |
| DS-50890 | 16090320 | 15828666 | 7696372 | 3588973 | 3456033 | 3044668 | 3792814 | 0.9463 | 0.9112 | 0.8027 | 151.653 |
| DS-50887 | 18410856 | 17841578 | 5586948 | 3592039 | 3463211 | 3080827 | 3792814 | 0.9471 | 0.9131 | 0.8123 | 167.1205 |
| DS-48649 | 11270170 | 11138367 | 3484190 | 3531251 | 3352098 | 2776824 | 3792814 | 0.931 | 0.8838 | 0.7321 | 103.6025 |
| DS-48662 | 20759492 | 20184756 | 7613229 | 3598912 | 3480999 | 3141637 | 3792814 | 0.9489 | 0.9178 | 0.8283 | 178.2999 |
| DS-50925 | 16170666 | 15755755 | 4935232 | 3590047 | 3450516 | 3025283 | 3792814 | 0.9465 | 0.9098 | 0.7976 | 147.2221 |
| DS-49502 | 19867392 | 19274375 | 13367481 | 3609224 | 3494888 | 3154283 | 3792814 | 0.9516 | 0.9214 | 0.8316 | 174.0566 |
| DS-68849 | 14392772 | 14207219 | 3560249 | 3577105 | 3438476 | 3016177 | 3792814 | 0.9431 | 0.9066 | 0.7952 | 145.0629 |
| DS-68877 | 17996088 | 17668724 | 4822556 | 3607513 | 3488074 | 3146684 | 3792814 | 0.9511 | 0.9197 | 0.8296 | 174.3006 |
| DS-56683 | 14558288 | 14217909 | 4688268 | 3552383 | 3403229 | 2949498 | 3792814 | 0.9366 | 0.8973 | 0.7777 | 134.6772 |
| DS-56691 | 19260558 | 18597047 | 10601226 | 3603747 | 3485037 | 3124138 | 3792814 | 0.9502 | 0.9189 | 0.8237 | 166.498 |
| DS-56626 | 13275722 | 13050437 | 4317444 | 3550964 | 3392247 | 2890678 | 3792814 | 0.9362 | 0.8944 | 0.7621 | 130.3596 |
| DS-49477 | 15768010 | 15212372 | 4742231 | 3549254 | 3391556 | 2918473 | 3792814 | 0.9358 | 0.8942 | 0.7695 | 137.9831 |
| DS-50283 | 16324092 | 15657652 | 11448352 | 3577910 | 3442383 | 3031429 | 3792814 | 0.9433 | 0.9076 | 0.7993 | 149.9973 |
| DS-48732 | 15177596 | 14617838 | 8007351 | 3546412 | 3394899 | 2944827 | 3792814 | 0.935 | 0.8951 | 0.7764 | 130.8796 |
|  |  |  |  |  |  |  |  |  |  |  |  |
| **Average** | 15979778 | 15562450 | 6389074 | 3538617 | 3371409 | 2913500 | 3792814 | 0.932982 | 0.888897 | 0.768161 | 144.3292 |
| **Median** | 16209684 | 15785209 | 5390906 | 3575976 | 3439106 | 3018231 | 3792814 | 0.94285 | 0.90675 | 0.79575 | 146.682 |

Reads_near_target: Number of sequence reads aligning to (target regions + 25 flanking bp)

Depth_ge10: Number of targeted bases with filtered depth greater than or equal to 10

Depth_ge20: Number of targeted bases with filtered depth greater than or equal to 20

Depth_ge50: Number of targeted bases with filtered depth greater than or equal to 50

**Table C. Mutation sequencing depth**

| **Sample Pair** | **Chromosome** | **Position** | **REF** | **ALT** | **Gene** | **AA change** | **Primary sample** | **Metastatic sample** | **Primary GT** | **Metastatic GT** | **Primary AF** | **Metastatic AF** | **Primary depth** | **Metastatic depth** |
| --- | --- | --- | --- | --- | --- | --- | --- | --- | --- | --- | --- | --- | --- | --- |
| A | 18 | 48575116 | C | T | SMAD4 | L104F | DS-50890* | DS-50887 | CT | nocall | 0.1079 | 0 | 167 | 263 |
| B | 7 | 14378155 | T | C | DGKB | K704E | DS-68849* | DS-68877 | TC | nocall | 0.156 | 0.03834 | 218 | 313 |
| C | 1 | 39893766 | G | A | MACF1 | S3475X | DS-50925 | DS-49502* | nocall | nocall | 0.01807 | 0.1564 | 166 | 211 |
| C | 1 | 198711429 | A | C | PTPRC | N714T | DS-50925 | DS-49502* | nocall | AC | 0 | 0.209 | 168 | 177 |
| D | 1 | 65307279 | CTCTT | C | JAK1 | 802_803_del | DS-53055* | DS-54174 | CTCTT:C | nocall | 0.321 | 0 | 78 | 97 |
| D | 5 | 86672805 | G | C | RASA1 | K587N | DS-53055* | DS-54174 | GC | nocall | 0.2079 | 0.005181 | 202 | 193 |
| D | 20 | 52198928 | G | C | ZNF217 | H146Q | DS-53055* | DS-54174 | GC | nocall | 0.1292 | 0 | 325 | 401 |
| D | 10 | 131557570 | G | A | MGMT | A158T | DS-53055 | DS-54174* | nocall | GA | 0.05 | 0.1818 | 20 | 44 |
| D | 20 | 47324917 | C | T | PREX1 | A222T | DS-53055 | DS-54174* | nocall | CT | 0 | 0.2049 | 98 | 244 |
| D | 11 | 67196610 | G | C | RPS6KB2 | E47Q | DS-53055 | DS-54174* | nocall | GC | 0 | 0.298 | 122 | 190 |
| D | 15 | 57574756 | G | A | TCF12 | E504K/E698K | DS-53055 | DS-54174* | nocall | GA | 0 | 0.3388 | 111 | 242 |
| D | 3 | 25665194 | G | A | TOP2B | L842F | DS-53055 | DS-54174* | nocall | GA | 0 | 0.2917 | 50 | 72 |
| D | 2 | 1426892 | C | T | TPO | T57M | DS-53055 | DS-54174* | nocall | CT | 0 | 0.2424 | 16 | 33 |
| E1 | 5 | 112116592 | C | T | APC | R223X/R213X | DS-51202* | DS-51211 | CT | nocall | 0.375 | 0.08929 | 72 | 56 |
| E1 | 2 | 212543874 | TG | T | ERBB4 | S508fs | DS-51202* | DS-51211 | TG:T | nocall | 0.2206 | 0 | 136 | 132 |
| E1 | 1 | 220702187 | C | T | MARK1 | P8L | DS-51202* | DS-51211 | CT | nocall | 0.6 | 0 | 5 | 8 |
| E1 | 2 | 179399472 | A | G | TTN | I24892T/I31389T | DS-51202* | DS-51211 | AG | nocall | 0.2424 | 0 | 198 | 142 |
| E1 | 11 | 4095746 | A | T | STIM1 | Q269L | DS-51202 | DS-51211* | nocall | AT | 0 | 0.2791 | 19 | 43 |
| E1 | 17 | 27076072 | G | A | TRAF4 | R297Q | DS-51202 | DS-51211* | nocall | GA | 0 | 0.2308 | 16 | 52 |
| E2 | 9 | 87317099 | G | T | NTRK2 | E80X | DS-59560* | DS-57659 | GT | nocall | 0.2192 | 0.07477 | 260 | 214 |
| E2 | 20 | 57829420 | A | C | ZNF831 | R1552S | DS-59560* | DS-57659 | AC | nocall | 0.1387 | 0.07143 | 238 | 210 |
| E2 | 4 | 87703368 | G | T | PTPN13 | G1974W/G1998W | DS-59560 | DS-57659* | nocall | GT | 0 | 0.1102 | 230 | 254 |
| E2 | 17 | 27076072 | G | A | TRAF4 | R297Q | DS-59560 | DS-57659* | nocall | GA | 0.02564 | 0.2857 | 39 | 49 |
| F | 6 | 44219918 | GAGA | G | HSP90AB1 | 549_550del | DS-48789* | DS-54095 | GAGA:G | nocall | 0.05645 | 0.00571 | 124 | 175 |
| F | 1 | 145537182 | T | C | ITGA10 | F785L | DS-48789* | DS-54095 | TC | nocall | 0.104 | 0 | 173 | 167 |
| F | 16 | 2339457 | G | A | ABCA3 | T893I | DS-48789* | DS-54095 | GA | nocall | 0.3158 | 0.06667 | 19 | 15 |
| F | 19 | 42796597 | T | C | CIC | F1052L | DS-48789 | DS-54095* | nocall | TC | 0 | 0.1579 | 29 | 57 |
| F | 4 | 66467560 | C | G | EPHA5 | V237L | DS-48789 | DS-54095* | nocall | CG | 0 | 0.1839 | 90 | 87 |
| F | 14 | 94158151 | G | A | KIAA1409/UNC79 | W2305X | DS-48789 | DS-54095* | nocall | GA | 0.06796 | 0.25 | 206 | 160 |
| F | 15 | 41989169 | A | G | MGA | K654R | DS-48789 | DS-54095* | nocall | AG | 0.05446 | 0.166 | 202 | 241 |
| F | 16 | 55536720 | C | A | MMP2 | P600H | DS-48789 | DS-54095* | nocall | CA | 0 | 0.1546 | 86 | 97 |
| F | 12 | 63195861 | C | T | PPM1H | R164H | DS-48789 | DS-54095* | nocall | CT | 0 | 0.1217 | 168 | 189 |
| F | 1 | 2116115 | G | A | PRKCZ | V374M/V557M | DS-48789 | DS-54095* | nocall | GA | 0.07246 | 0.1988 | 138 | 171 |
| F | 19 | 4175882 | G | A | SIRT6 | R164W | DS-48789 | DS-54095* | nocall | GA | 0 | 0.1731 | 43 | 52 |
| F | 7 | 17843106 | A | G | SNX13 | M721T | DS-48789 | DS-54095* | nocall | AG | 0 | 0.2174 | 247 | 299 |
| F | 19 | 1619215 | CG | C | TCF3 | P448fs | DS-48789 | DS-54095* | nocall | CG:C | 0.08333 | 0.4359 | 24 | 39 |
| F | 7 | 116955376 | C | T | WNT2 | A113T | DS-48789 | DS-54095* | nocall | CT | 0 | 0.1678 | 142 | 143 |
| F | 17 | 41607535 | C | T | ETV4 | A276T | DS-48789 | DS-54095* | nocall | CT | 0.08065 | 0.1507 | 62 | 73 |
| H | 17 | 7578263 | G | A | TP53 | R196X | DS-56683 | DS-56691* | nocall | GA | 0.03509 | 0.4177 | 57 | 79 |
| H | 15 | 40917810 | A | C | CASC5 | K1809T | DS-56683 | DS-56691* | nocall | AC | 0.08065 | 0.2468 | 62 | 77 |
| H | 1 | 6194315 | G | A | CHD5 | A1006V | DS-56683 | DS-56691* | nocall | GA | 0.08929 | 0.4205 | 56 | 88 |
| H | 4 | 153251907 | G | A | FBXW7 | R249X | DS-56683 | DS-56691* | nocall | GA | 0.02299 | 0.2794 | 87 | 68 |
| H | 12 | 123107032 | TATC | T | KNTC1 | 2132_2132del | DS-56683 | DS-56691* | nocall | TATC:T | 0.04854 | 0.2474 | 103 | 97 |
| H | 5 | 112175147 | G | T | APC | E1268X | DS-56683 | DS-56691* | nocall | GT | 0.02778 | 0.2093 | 72 | 86 |
| I | 17 | 7577058 | C | CA | TP53 | E294fs | DS-50283* | DS-48732 | C:CA | nocall | 0.2877 | 0 | 358 | 324 |
| I | 13 | 32944645 | G | T | BRCA2 | G2813V | DS-50283* | DS-48732 | GT | nocall | 0.2255 | 0 | 102 | 110 |
| I | 3 | 39307607 | C | T | CX3CR1 | V132I/V164I | DS-50283* | DS-48732 | CT | nocall | 0.1018 | 0 | 167 | 129 |
| I | 1 | 39893233 | G | C | MACF1 | T3413P/E3413Q | DS-50283* | DS-48732 | GC | nocall | 0.2176 | 0.04598 | 205 | 174 |
| I | 7 | 78150821 | G | A | MAGI2 | A227V | DS-50283* | DS-48732 | GA | nocall | 0.3263 | 0 | 426 | 354 |
| I | 18 | 45368209 | T | A | SMAD2 | S465C | DS-50283* | DS-48732 | TA | nocall | 0.3604 | 0 | 111 | 157 |
| I | 15 | 67358643 | C | G | SMAD3 | L51V | DS-50283* | DS-48732 | CG | nocall | 0.3235 | 0 | 34 | 37 |
| I | 6 | 152640069 | C | A | SYNE1 | D5440Y | DS-50283* | DS-48732 | CA | nocall | 0.2762 | 0 | 181 | 160 |
| I | 8 | 9592494 | CAAG | C | TNKS | 812_812del | DS-50283* | DS-48732 | CAAG:C | nocall | 0.3361 | 0 | 122 | 153 |
| I | 7 | 116955193 | CATCCACAAATGCGCGGGCAAATTTG | C | WNT2 | 165_173del | DS-50283* | DS-48732 | CATCCACAAATGCGCGGGCAAATTTG:C | nocall | 0.06849 | 0 | 292 | 268 |
| I | 2 | 179616266 | C | T | TTN | E3621K | DS-50283* | DS-48732 | CT | nocall | 0.2206 | 0 | 68 | 66 |
| I | 3 | 122423428 | A | C | PARP14 | K1125Q | DS-50283* | DS-48732 | AC | nocall | 0.3237 | 0.07857 | 173 | 140 |
| I | 7 | 45719364 | T | G | ADCY1 | V652G | DS-50283 | DS-48732* | nocall | TG | 0 | 0.09424 | 318 | 191 |
| I | 5 | 66459227 | C | G | MAST4 | S1407C | DS-50283 | DS-48732* | nocall | CG | 0.0505 | 0.1742 | 99 | 132 |
| I | 12 | 117662898 | A | T | NOS1 | I1250N/I1284N | DS-50283 | DS-48732* | nocall | AT | 0.06081 | 0.1603 | 148 | 131 |
| J | 17 | 56688600 | C | T | TEX14 | R369H/R375H | DS-48649* | DS-48662 | CT | nocall | 0.1064 | 0.07241 | 141 | 290 |
| K | 7 | 106508805 | G | A | PIK3CG | E267K | DS-48595* | DS-59982 | GA | nocall | 0.3209 | 0 | 187 | 217 |
| K | 3 | 78685010 | C | T | ROBO1 | E1096K | DS-48595* | DS-59982 | CT | nocall | 0.2917 | 0.07964 | 384 | 226 |
| K | 4 | 87023078 | T | G | MAPK10 | K140T/K178T | DS-48595 | DS-59982* | nocall | TG | 0 | 0.1073 | 202 | 177 |
| L | 8 | 114186005 | T | A | CSMD3 | I179L/I219L | DS-44604 | DS-67176* | nocall | TA | 0 | 0.1495 | 18 | 107 |
| L | 15 | 64039944 | G | A | HERC1 | P778L | DS-44604 | DS-67176* | nocall | GA | 0 | 0.3273 | 15 | 55 |
| L | 19 | 9064300 | G | T | MUC16 | P7716T | DS-44604 | DS-67176* | nocall | GT | 0 | 0.2128 | 18 | 94 |
| L | 9 | 8485280 | C | T | PTPRD | V613M/V1043M | DS-44604 | DS-67176* | nocall | CT | 0 | 0.2073 | 9 | 82 |
| M | 16 | 30515574 | G | T | ITGAL | D742Y | DS-52992* | DS-54373 | GT | nocall | 0.1833 | 0 | 60 | 83 |
| N | 9 | 35077312 | C | G | FANCG | D199H | DS-54072 | DS-53085* | nocall | CG | 0 | 0.1954 | 326 | 389 |
| O | 13 | 92346125 | T | A | GPC5 | L337X | DS-50851* | DS-59918 | TA | nocall | 0.1682 | 0 | 214 | 202 |
| O | 8 | 17827238 | A | G | PCM1 | I1189V | DS-50851* | DS-59918 | AG | nocall | 0.2 | 0.03226 | 60 | 31 |
| O | 14 | 74824969 | G | A | VRTN | G495R | DS-50851* | DS-59918 | GA | nocall | 0.4194 | 0 | 31 | 37 |
| O | 16 | 77356260 | AT | A | ADAMTS18 | N712fs | DS-50851 | DS-59918* | nocall | AT:A | 0 | 0.3111 | 138 | 180 |
| P | 12 | 43828166 | G | A | ADAMTS20 | R868X | DS-56626* | DS-49477 | GA | nocall | 0.1953 | 0 | 215 | 241 |
| P | 2 | 140992404 | G | A | LRP1B | A4537V | DS-56626* | DS-49477 | GA | nocall | 0.1471 | 0 | 136 | 182 |
| P | 6 | 137112932 | T | A | MAP3K5 | T122S | DS-56626* | DS-49477 | TA | nocall | 0.1946 | 0 | 149 | 126 |
| Q | 1 | 43805205 | C | G | MPL | Q219E | DS-48725 | DS-48634* | nocall | CG | 0.0619 | 0.2329 | 339 | 292 |
| Q | 4 | 104510991 | T | A | TACR3 | N416Y | DS-48725 | DS-48634* | nocall | TA | 0.0579 | 0.2297 | 432 | 418 |
| R | 2 | 179427560 | G | T | TTN | P18702T | DS-55324* | DS-55353 | GT | nocall | 0.3443 | 0.02778 | 61 | 72 |
| R | 2 | 179485946 | C | T | TTN | V125991I/V6102I | DS-55324* | DS-55353 | CT | nocall | 0.2833 | 0.05159 | 293 | 504 |
| R | 1 | 147090949 | GC | G | BCL9 | A330fs | DS-55324 | DS-55353* | nocall | GC:G | 0 | 0.1551 | 57 | 58 |
| R | 12 | 43763099 | G | C | ADAMTS20 | C1844W | DS-55324 | DS-55353* | nocall | GC | 0.0303 | 0.1622 | 33 | 37 |
| R | 3 | 48618070 | G | A | COL7A1 | R1666W | DS-55324 | DS-55353* | nocall | GA | 0.05405 | 0.1892 | 37 | 37 |
| R | 5 | 86675592 | C | G | RASA1 | T843S/T666S | DS-55324 | DS-55353* | nocall | CG | 0 | 0.1802 | 72 | 111 |
| R | 8 | 53574204 | C | T | RB1CC1 | A417T | DS-55324 | DS-55353* | nocall | CT | 0.0303 | 0.2485 | 99 | 165 |
| R | 3 | 78717053 | G | T | ROBO1 | S649X | DS-55324 | DS-55353* | nocall | GT | 0.05622 | 0.1848 | 249 | 433 |
| R | 3 | 133356863 | C | T | TOPBP1 | A793T | DS-55324 | DS-55353* | nocall | CT | 0.0631 | 0.21 | 206 | 281 |
| R | 2 | 179616674 | C | G | TTN | A3485P | DS-55324 | DS-55353* | nocall | CG | 0.05941 | 0.1503 | 101 | 153 |

GT: Genotype

AF: Allele frequency

Note that the “primary/met” designation does not apply to sample pairs L and O, as both samples are metastatic.

The asterisk indicates the sample with the mutation.
